# A chromosome-scale genome and transcriptomic analysis of the endangered tropical tree *Vatica mangachapoi* (Dipterocarpaceae)

**DOI:** 10.1093/dnares/dsac005

**Published:** 2022-02-16

**Authors:** Liang Tang, Xuezhu Liao, Luke R Tembrock, Song Ge, Zhiqiang Wu

**Affiliations:** 1 Center for Terrestrial Biodiversity of the South China Sea, Hainan University, Haikou, Hainan 570228, China; 2 Shenzhen Branch, Guangdong Laboratory for Lingnan Modern Agriculture, Genome Analysis Laboratory of the Ministry of Agriculture, Agricultural Genomics Institute at Shenzhen, Shenzhen 518120, China; 3 Department of Agricultural Biology, Colorado State University, Fort Collins, CO 80523, USA; 4 State Key Laboratory of Systematic and Evolutionary Botany, Institute of Botany, Chinese Academy of Sciences, Beijing 100093, China

**Keywords:** dipterocarp forests, genome assembly, whole-genome duplication, tree genomics, conservation biology

## Abstract

*Vatica mangachapoi* is a tropical tree species native to Southeast Asia. It has long been valued as a timber species because the wood resists decay, but it is now considered vulnerable to extinction due to habitat loss and overexploitation. Here, we present the first chromosome-level genome assembly of *V. mangachapo*i that we created by combining data from PacBio long read sequencing with Hi-C proximity ligation and Illumina short-read sequencing. The assembled genome was 456.21 Mb, containing 11 chromosome and a BUSCO score of 93.4%. From the newly assembled genome, 46,811 protein-coding genes were predicted. Repetitive DNA accounted for 53% of the genome. Phylogenomic and gene family analyses showed that *V. mangachapoi* diverged from a common ancestor of *Gossypium raimondii* 70 million years ago. Transcriptome analyses found 227 genes that were differentially expressed in the leaves of plants grown in normal soil relative to plants grown in dry, coastal, sandy soil. For these genes, we identified three significantly enriched with GO terms: responses to organonitrogen compounds, chitin-triggered immunity, and wound response. This genome provides an important comparative benchmark not only for future conservation work on *V. mangachapoi* but also for phylogenomics work on Dipterocarpaceae.

## 1. Introduction

Dipterocarpaceae is a pantropically distributed family of trees known for producing high-value timber and for being a species of ecological importance, including ∼500 species. Dipterocarpoideae is the largest and most diverse subfamily, comprising 13 genera and accounting for over 90% of the species in the family.^1^ Species of the Dipterocarpoideae provides the foundation on the establishment of ecosystems in tropical forests.^2^ In Southeast Asian tropical forests, Asian dipterocarp forests provide a variety of ecosystem services, including global carbon balance, regional climate regulation, and watershed services. However, Southeast Asia has experienced rapid deforestation and biodiversity loss in recent decades.^3^ Indeed, the unsustainable exploitation of dipterocarp forests for timber resources has led to a massive loss of tropical forest land in Southeast Asia. Consequently, many species in Dipterocarpaceae are currently classified as threatened or even critically endangered.^2^ Hence, protecting dipterocarp forests in Southeast Asia is crucial for climate change mitigation, the sustainable development of local communities, and the conservation of species that rely on these forests. In addition, a solid phylogenetic framework of the Dipterocarpaceae is required to resolve the origin, assembly process, and history of dipterocarp-dominated tropical forests.^4^ While considerable progress has been made recently in resolving the phylogenetic relationships among Dipterocarpaceae,^5^^,^^6^ further work is needed to identify orthologous genes, especially since some of these dipterocarp species originated through hybridization and polyploidization.^6^

In the past decade, genomic technologies have been increasingly applied to problems in conservation biology and genetics.^7^^,^^8^ With whole-genome data, the genetic sources of local adaptation across populations can be comprehensively quantified.^9–11^ Furthermore, mapping the genetic load and predicting inbreeding depression in species with low population sizes is vastly improved with complete genome data.^7^^,^^8^^,^^12^ Lastly, the completion of high-quality, chromosome-scale genomes can provide markers for species with fewer genomic resources.^13^ Thus, the complete genome sequences of Dipterocarpaceae species can aid in the conservation of endangered dipterocarp species and facilitate restoration of degraded Asian tropical forest ecosystems where they were once ecologically dominant.^14^^,^^15^

Recent advancements in sequencing, namely massive parallel short-read sequencing of Hi-C libraries combined with single-molecule long-read sequencing, have facilitated the assembly of accurate and near complete chromosome-level *de novo* genomes of species with little to no available genomic data.^16^ In this study, Illumina and PacBio sequencing were used in conjunction with Hi-C proximity ligation libraries to assemble a chromosome-level genome of *Vatica mangachapoi*, a species under second class protection in China once distributed throughout seasonal tropical rainforests from Borneo to Hainan Island, China. In addition to the completion of a high-quality genome, RNA-seq was used to quantify and characterize differences in gene expression between normal and water-stressed conditions in this species. The high-quality genome of *V.mangachapoi* will serve as a reference to study fundamental gene expression pathways in large tropical trees such as flooding tolerance, wood formation, and long-term/seasonal environmental adaptations.^15^^,^^17^ From this, more efficient conservation strategies can be deployed such as identifying and planting adequately adapted genotypes in deforested areas and reducing inbreeding depression through planned pedigree mating.^7^^,^^14^ Here, we present a chromosome-level genome assembly of *V. mangachapoi* and provide new insights into the evolutionary history and the genetic mechanisms behind drought resistance for this species using transcriptomic data.

## 2. Materials and methods

### 2.1. Sample collection and genome sequencing

Tissue from *V. mangachapoi* Blanco was collected from Jinniuling Park in Hainan, Chin, Haikou, China for whole-genome sequencing. High-quality DNA was extracted from fresh leaves by using QIAGEN^®^ Genomic kits and the DNA quantification was checked by Nanodrop and Qubit. Five Illumina paired-end libraries with insertion sizes of 250 bp, 450 bp, 2 kb, 5 kb, and 10 kb were sequenced on an Illumina HiSeq platform to generate whole-genome shotgun data using the Illumina standard methods (San Diego, USA). A 15 kb DNA SMRT Bell library was generated to sequence the genome on a PacBio Sequel2 platform. The Hi-C libraries were generated using standard procedures^18^^,^^19^ and sequenced on an Illumina HiSeq X platform to generate paired-ends reads. Seven *V. mangachapoi* samples were used to generate RNA-seq data, including four drought-stressed leaves collected from plants grown in coastal sandy substrate (sampled from the *V. mangachapoi* Provincial Natural Reserve in Hainan, China) and three leaf samples collected from plants grown in normal soil (sampled from the Xinglong Botanical Garden and from Jinniuling Park in Hainan, China). Total RNA was extracted from various plant organs (roots, leaves and young fruit), and residual DNA was removed by using RNAprep pure Plant Kit (TIANGEN) and then 150 bp paired-end libraries were generated and sequenced on an Illumina HiSeq platform.

### 2.2. Genome survey and assembly

The quality of raw reads was evaluated using FastQC v 0.11.7 (http://www.bioinformatics.babraham.ac.uk/projects/fastqc/), and poor-quality reads were trimmed using Trimmomatic v0.38.^20^ A total of 57 Gb of clean Illumina reads were produced by Illumina paired-end sequencing and to conduct a genome survey with Jellyfish v2.1.4^21^ and Genomescope2.^22^ The genome size, heterozygosity, and repeat content were estimated according to K-mer frequency distributions (K-mer = 21). Three long-reads assembly strategies (Canu, Canu plus Flye, and Falcon) were tested on the 84.81 Gb PacBio CLR reads.^23^ The best assembly was given to Racon^24^ for three runs of correction. Pilon^25^ was then employed to carry out one correction step on the Illumina data. BUSCO v4.0.6^26^ in conjunction with the embryophyta_odb10 database was used to assess the quality of genome assembly and completeness of the annotation.

### 2.3. Chromosome assembly using Hi-C data

A 150 bp paired-end Hi-C library was sequenced on an Illumina HiSeq X platform, producing 103.45 Gb of high-quality (Q20 ≥ 96.67%) sequencing data. Juicer v1.5.6^27^ and HiC-Pro v2.10.0^28^ were used to map the reads to the assembled genome and assess the quality of the Hi-C library. 3D-DNA^29^ software was run to divide, rank, and orient the genome sequences and to evaluate the assembled genome. The chromosome-level assembly of the *V. mangachapoi* genome was visualized with HiCPlotter.^30^

### 2.4. Identification of repetitive elements

Repeats were identified using homolog-based and *de novo* prediction methods. RepeatMasker v4.0.7^31^ and RepeatProteinMask v4.0.7^31^ were conducted to identify repetitive sequences based on homology to known repeats deposited in RepBase v21.12 (http://www.girinst.org/repbase). RepeatModeler (http://www.repeatmasker.org/RepeatModeler/), a software package based on RepeatScout,^32^ and Tandem Repeats Finder v4.09 (http://tandem.bu.edu/trf/trf.html), was used to identify repeats *ab initio* and to build a repeat library. In addition, LTR retrotransposons were identified using LTR_FINDER v1.06 (http://tlife.fudan.edu.cn/ltr_finder/). RepeatMasker v4.0.7^31^ integrated all of the repeats identified above to generate a final repeat annotation file.

### 2.5. Protein-coding gene prediction and functional annotation

Three strategies (gene homology, *de novo* gene prediction, and transcriptional evidence) were combined to accurately predict protein-coding genes in *V. mangachapoi*. Gene structure was first predicted by Genewise v2.4.1 (https://www.ebi.ac.uk/Tools/psa/genewise/) based on protein sequences from *Theobroma cacao* (PRJEB14326), *Gossypium arboreum* (PRJNA335838), *Prunus mume* (PRJNA246160), *Prunus avium* (PRJDB4877), *Prunus armeniaca* (PRJEB37669), and *Amygdalus communis* (PRJNA631757) derived from NCBI. Then, *de novo* prediction of gene structure was performed using Augustus.^33^ RNA extracted from leaves, inflorescences, and immature fruits was used to generate transcriptomic data. The reads were mapped with Hisat2 (https://daehwankimlab.github.io/hisat2/) and assembled with Cufflinks (http://cole-trapnell-lab.github.io/cufflinks/). A complete and non-redundant gene set was created by integrating annotations obtained from the above three methods into the software tool Maker (https://weatherby.genetics.utah.edu/MAKER/wiki/index.php/MAKER_Tutorial_for_WGS_Assembly_and_Annotation_Winter_School_2018).

Functional annotation was performed using eggNOG-mapper^34^ which is a tool that predicts gene function based on fast orthology assignments. iTAK^35^ was used to predict transcription factors (TFs) in *V. mangachapoi* and other eight species (*Solanum lycopersicum*, *Gossypiumraimondii*, *Oryza sativa*, *Populus trichocarpa*, *Vitis vinifera*, *Camellia sinensis*, *Solanum tuberosum*, and *Arabidopsis thaliana*) using the PlantTFDB database (http://planttfdb.gao-lab.org/).

### 2.6. Identification of noncoding-RNA genes

We used tRNAscan-SE v1.3.1 (http://lowelab.ucsc.edu/tRNAscan-SE/)^36^ to identify tRNA genes. The program INFERNAL^37^ was carried out with default parameters to annotate snRNAs and miRNAs in the assembled genome of *V. mangachapoi*.

### 2.7. Phylogenetic analysis

Protein-coding orthologs from *V. mangachapoi* and eight other high-quality genomes (*S. lycopersicum*, *S. tuberosum*, *V. vinifera*, *A. thaliana*, *C. sinensis*, *P. trichocarpa*, *G. raimondii*, and *O. sativa*) were extracted by Orthofinder v2.3.3.^38^ According to the results of gene family clustering, 779 single-copy gene families were picked out and the corresponding multi-sequence alignments were generated using Muscle v3.8.31.^39^ The phylogenetic tree was inferred using RAxML v8^40^ and visualized in FigTree v1.4.3 (http://tree.bio.ed.ac.uk/software/figtree/). The CODEML and MCMCTREE programs from the PAML v4.5^41^ software package were used to estimate the substitution rate and divergence times, respectively. Calibration points retrieved from TimeTree (http://timetree.org/) were used as priors in divergence time estimation.

### 2.8. Expansion and contraction of gene families

Expansion and contraction of gene families were analysed in relation to the time-calibrated phylogeny of the nine species (Fig. 2). We discarded gene families with >200 members. In addition, gene families in which each species had at least one family member were kept for analysis. The CAFE v4.2.1^42^ (Computational Analysis of Gene Family Evolution) program was used to detect expansion and contraction in gene families. GO enrichment was carried out using the R package ClusterProfiler^43^ on gene families undergoing significant expansion and contraction. With these GO annotations, an OrgDb database was constructed for *V. mangachapoi*.

### 2.9. Analysis of synteny and whole-genome duplication

Homologous proteins in *G. raimondii* and *V. mangachapoi* were identified with BLASTP^44^ (E-value = 1e−5), and collinear blocks were detected using MCScanX.^45^ Collinearity within *V. mangachapoi* and between the two species was analysed using JCVI v0.8.12.^46^ Collinear genes were aligned and the Ks of each gene pair was calculated using CODEML in PAML v4.5.^41^ Changes in the effective population sizes of *V. mangachapoi* over time were modelled using PSMC v0.6.5-r67 (https://github.com/lh3/psmc). The neutral mutation rate was estimated to be roughly $4.77\;\times \;10^{-9}$ on basis of the Ks and the time-calibrated phylogeny (Fig. 2). In addition, an average generation time of 25 yrs was assumed.

### 2.10. Transcriptome analysis and identification of highly expressed genes

RNA-seq was performed on seven leaf samples. This generated raw reads that were filtered using Trimmomatic v0.39.^20^ These cleaned data (8–11 Gb per sample) were mapped to the reference genome using STAR.^47^ The expression level of each gene was calculated using featureCounts v2.0.1,^48^ and differential expression was analysed using Deseq2.^49^ The genes were kept if ‘*P*-adjust < 0.05 and |log2 Fold Change| >1’. Functional enrichment analysis was carried out on differentially expressed genes using Clusterprofiler.^43^

### 2.11. Phylogenetic analysis of *TPS* gene family

Known *TPS* genes in *A. thaliana*^50^ (*TPS*-a: *AT4G15870*, *AT2G23230*, *AT1G70080*, *AT4G20200*, *AT4G20210*, *AT4G20230*, *AT5G44630*, *AT4G13280*, *AT4G13300*, *AT3G29190*, *AT3G29110*, *AT3G14490*, *AT3G14520*, *AT3G14540*, *AT5G48110*, *AT5G23960*, *AT1G33750*, *AT3G29410*, *AT1G66020*, *AT1G48800*, *AT1G31950*, *AT3G32030*, *AT1G48820*; *TPS*-b: *AT4G16730*, *AT4G16740*, *AT2G24210*, *AT3G25830*, *AT3G25810*, *AT3G25820*; *TPS*-c: *AT4G02780*; *TPS*-e: *AT1G79460*; *TPS*-f: *AT1G61120*; *TPS*-g: *AT1G61680*) were used as queries to identify potential *TPS* genes encoded by *V. mangachapoi* during BLASTP^44^ searches. The amino acid sequences of the *TPS* genes in both species were aligned using MAFFT v7.310.^51^ The alignment was trimmed using trimAl v1.4.rev15,^52^ and a maximum likelihood phylogeny was inferred from the best amino acid substitution model (JTT+F+R4) using IQTREE2.^53^

## 3. Results

### 3.1. Sequencing and assembly of the *V. mangachapoi* genome

**Figure 1:**
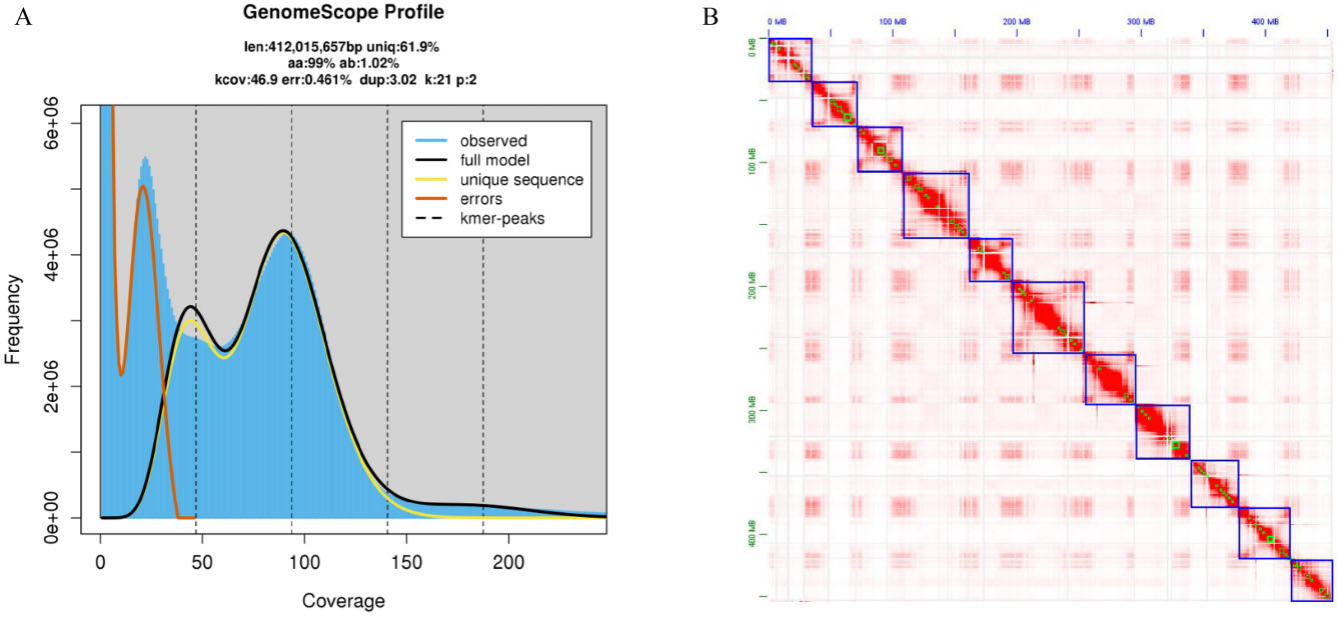
Characterization of the *V. mangachapoi* genome. (A) Frequency distribution of 21-mers derived from 57 Gb of cleaned Illumina sequencing reads. (B) Intensity signal heatmap of the Hi-C chromosome.

The genomic size of *V. mangachapoi* was estimated to be 392.30–434.77 Mb based on a K-mer analysis (Fig. 1A;Supplementary Table S1). The 21-mer distribution showed two peaks. Based on this, the level of heterozygosity in the genome was estimated to be ∼1.02% (Fig. 1A;Supplementary Table S1). To obtain a high-quality genome of *V. mangachapoi*, three long-reads assembly strategies (Canu, Canu plus Flye, and Falcon) were used to assemble 84.81 Gb of PacBio sequencing reads. The best result was obtained by Falcon, which produced an assembly with a total size of 582.79 Mb and a contig N50 size of 4.05 Mb. After Illumina reads were used for correction and 103.45 Gb high-quality Hi-C sequencing data was generated to help constructing a chromosome-level genome assembly by providing relationships and directions between sequences and removing redundant sequences. After manual correction, a genome assembly with 11 chromosomes was 456.21 Mb (Fig. 1B), containing 0.10% N sequences. The integrity of the assembled genome was assessed with BUSCO analysis using 1,614 conserved plant proteins. The results indicated that 93.4% of the total genes were identified in the annotation of *V. mangachapoi*, of which 73.6% were single copy and 8.9% were duplicated, therefore, this version of the genome is used for all subsequent analyses.

### 3.2. Genome annotations

The genome of *V. mangachapoi* was found to contain 243.3 Mb of repetitive DNA sequences, accounting for 53.33% of its genome. Among these repetitive sequences, LTR retrotransposons were the dominant type, making up of 179.95 Mb or 39.44% of the genome. *Gypsy* type LTRs totalled 113.66 Mb or 24.91% of the genome while *Copia* type LTRs totalled 38.11 Mb or 8.35% of the genome. Non-LTR retrotransposons, including LINEs and SINEs, made up a small portion of the genome, accounting for only 1.28% and 0.02% of the genome, respectively. Moreover, 18.02 Mb of DNA Transposons (class II TEs) were identified, accounting for 3.95% of the completed genome (Supplementary Table S2).

By combining transcriptome, homology, and *ab initio* gene prediction methods, we identified a total of 46,811 protein-coding genes, nearly half of which were responsible for encoding over 100 amino acids. Over half of the identified genes (26,369) with fragments per kilobase of transcript per million (FPKM) values >0.01 in at least one RNA-seq sample were transferred to a core gene set and employed in downstream analyses. Based on the core gene set, the mean length of protein-coding genes in *V. mangachapoi* was 3,892.1 bp, and each gene contained 7.24 exons, on average (Supplementary Table S3). Using three databases (COG, GO, and KEGG), functional annotation of the core gene set was performed, and 90.97% of the genes in the core gene set were annotated. About 90.60% of the genes had orthologs in COG, 43.45% had GO terms, and 51.50% were mapped to the known plant biological pathways in KEGG (Supplementary Table S4). BUSCO analysis showed that 87.7% of the conserved plant genes were present in our annotations (Supplementary Table S5).

TFs are ubiquitous elements of genomes and play important roles in plant development and environmental responses by regulating gene expression. Here, we identified a total of 2,289 TFs in *V. mangachapoi*, with the five largest families being *MYB* (192), *C2H2* (153), *AP2/ERF-ERF* (150), *BHLH* (144), and *NAC* (131) (Supplementary Table S6). Additionally, non-coding RNAs in the genome were identified and annotated, including 208 miRNAs, 1,737 rRNAs, 1,082 tRNAs, and 465 snRNAs (Supplementary Table S7).

### 3.3. Evolutionary history and whole-genome duplication

**Figure 2:**
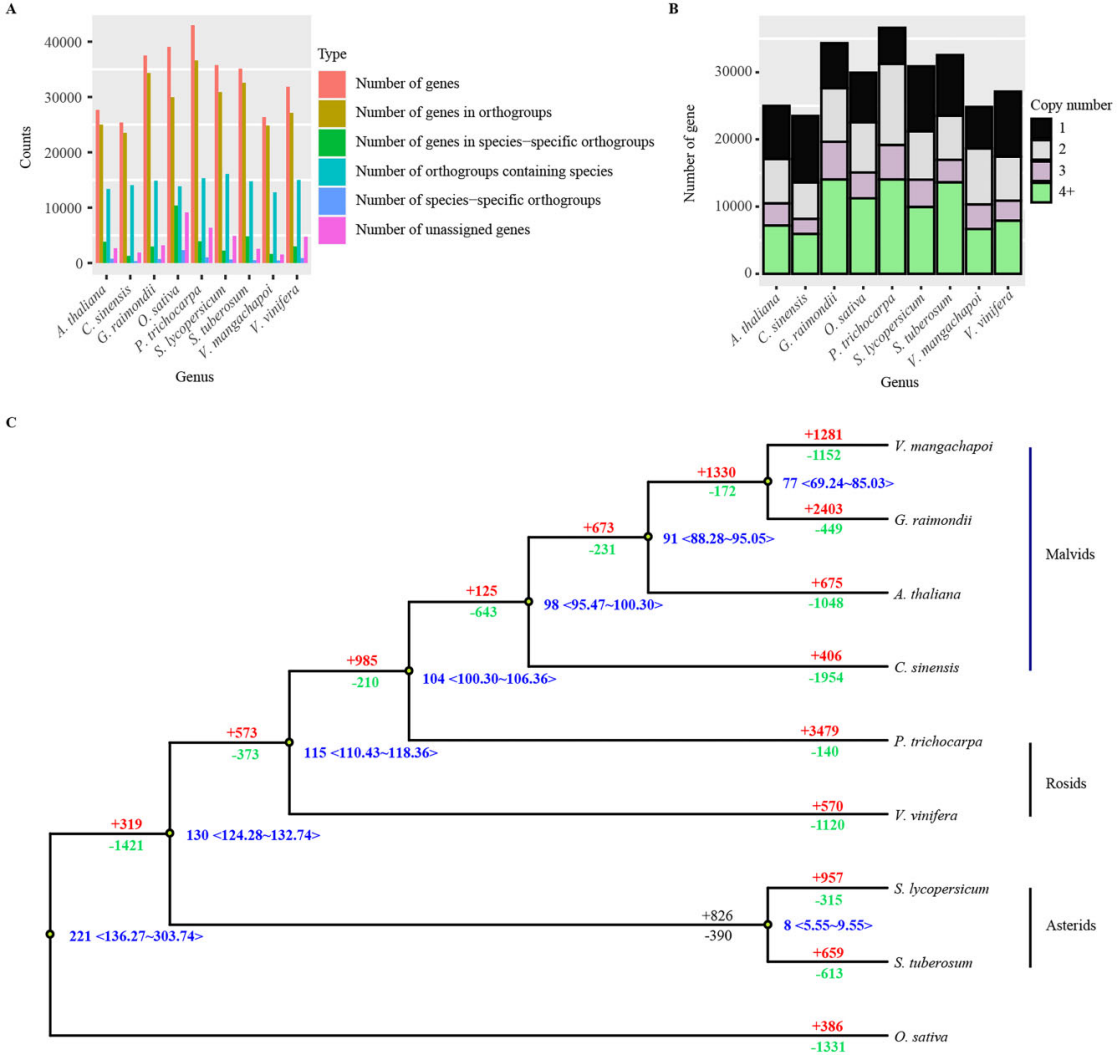
Comparison of protein-coding genes and phylogenetic analysis. (A) Comparison of classifications of protein-coding genes in the nine species used for comparative genomic analysis. (B) Copy number distribution of gene families in the nine species. (C) Phylogenetic tree inferred from the orthologous gene sets of *V. mangachapoi* and eight other species. The numbers after ‘+’ and ‘−’ represent the numbers of expanded or contracted gene families, respectively. Blue numbers indicate the estimated divergence times from MYA with 95% confidence intervals (CIs).

Phylogenetic analysis was carried out to study the evolutionary history of *V. mangachapoi*. Eight other species with whole-genome sequences, including three malvid species and five more distantly related species (Fig. 2C) were chosen for the analyses. The longest protein sequence available for each gene was chosen from each species for clustering with Orthofinder v2.3.3 to construct orthologous gene sets. A total of 24,840 genes from the core gene set of *V. mangachapoi* were clustered into orthogroups (Fig. 2A;Supplementary Table S8). Interestingly, there were fewer multi-copy genes in *V. mangachapoi* than in other species (Fig. 2B;Supplementary Table S9). We identified 779 high-quality, single-copy genes from the nine plant genomes and used them to reconstruct their phylogeny. The phylogenetic analysis showed that *V. mangachapoi* was most closely related to cotton as was expected based on results from APG IV (Fig. 2C).

Analysis of the expansion and contraction of gene families revealed that 1,281 and 1,152 gene families expanded and contracted in *V. mangachapoi*, respectively (Fig. 2C). According to the GO enrichment results, the expanded gene families were mainly related to amino acid metabolism, organic compound synthesis, and proteolysis, whereas the contracted gene families were associated with carbohydrate synthesis, cell growth and regulation, and the photopigment pathway (Supplementary Figs S1 and S2).

**Figure 3:**
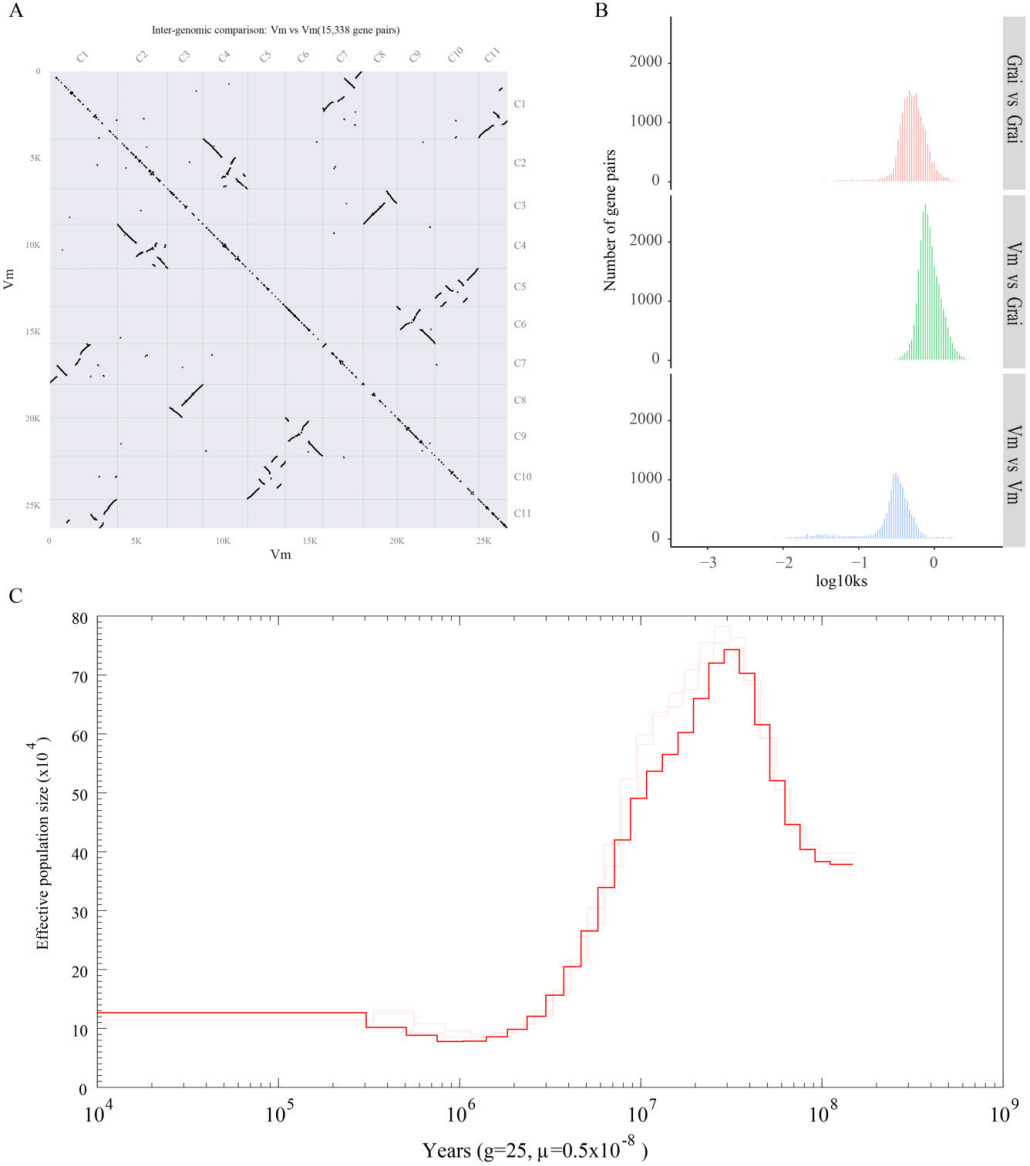
(A) Collinearity within the genome of *V. mangachapoi*. (B) Density distribution of Ks values between homologous pairs. Vm: *V. mangachapoi*, Grai: *G. raimondii*. (C) Separate and average (in red) historical population sizes of three *V. mangachapoi* populations. *g*, generation time; *μ*, substitution rate.

To analyse collinearity in the *V. mangachapoi* genome, BLASTP and MCScanX were conducted to identify homologous proteins. This analysis found 8,075 paralogous protein pairs within 395 colinear blocks in the genome of *V. mangachapoi*. Through comparative genomic analyses, we found that the chromosomes of *V. mangachapoi* were highly colinear with each other when inversions of large chromosomal segments were considered. Such collinearity suggested whole-genome duplication (WGD) have occurred in this species (Fig. 3A). The comparison of collinearity within the cotton genome and between the *V. mangachapoi* and cotton genomes both identified a large number of collinear blocks, consistent with the finding that cotton has undergone a WGD event^54^ (Supplementary Figs S3 and S4). The distribution of Ks within a genome can be used to detect WGD and estimate the time of such an event. For *V. mangachapoi*, WGD may have occurred 31.77 million years ago (MYA), whereas an independent WGD may have occurred in cotton ∼44.30 MYA. The divergence point of *V. mangachapoi* and cotton was estimated to have happened 69.24–85.03 MYA, consistent with the estimation given by http://timetree.org/. Therefore, after the divergence of *V. mangachapoi* and cotton, two independent WGD events may have occurred in these two lineages (Fig. 3B). The WGD detected in the *V. mangachapoi* genome and protein data sets explains the relatively high level of duplication (8.9% and 12%) in the BUSCO analysis. In addition, PSMC analyses revealed that three *V. mangachapoi* populations had similar effective population sizes that their peak effective population size reached was 750,000 individuals, and that these populations reached this size ∼20 MYA. After this, the population size gradually reduced down to 130,000 individuals (Fig. 3C).

### 3.4. RNA-seq analysis provides mechanistic insight into the drought tolerance of *V. mangachapoi*

**Figure 4:**
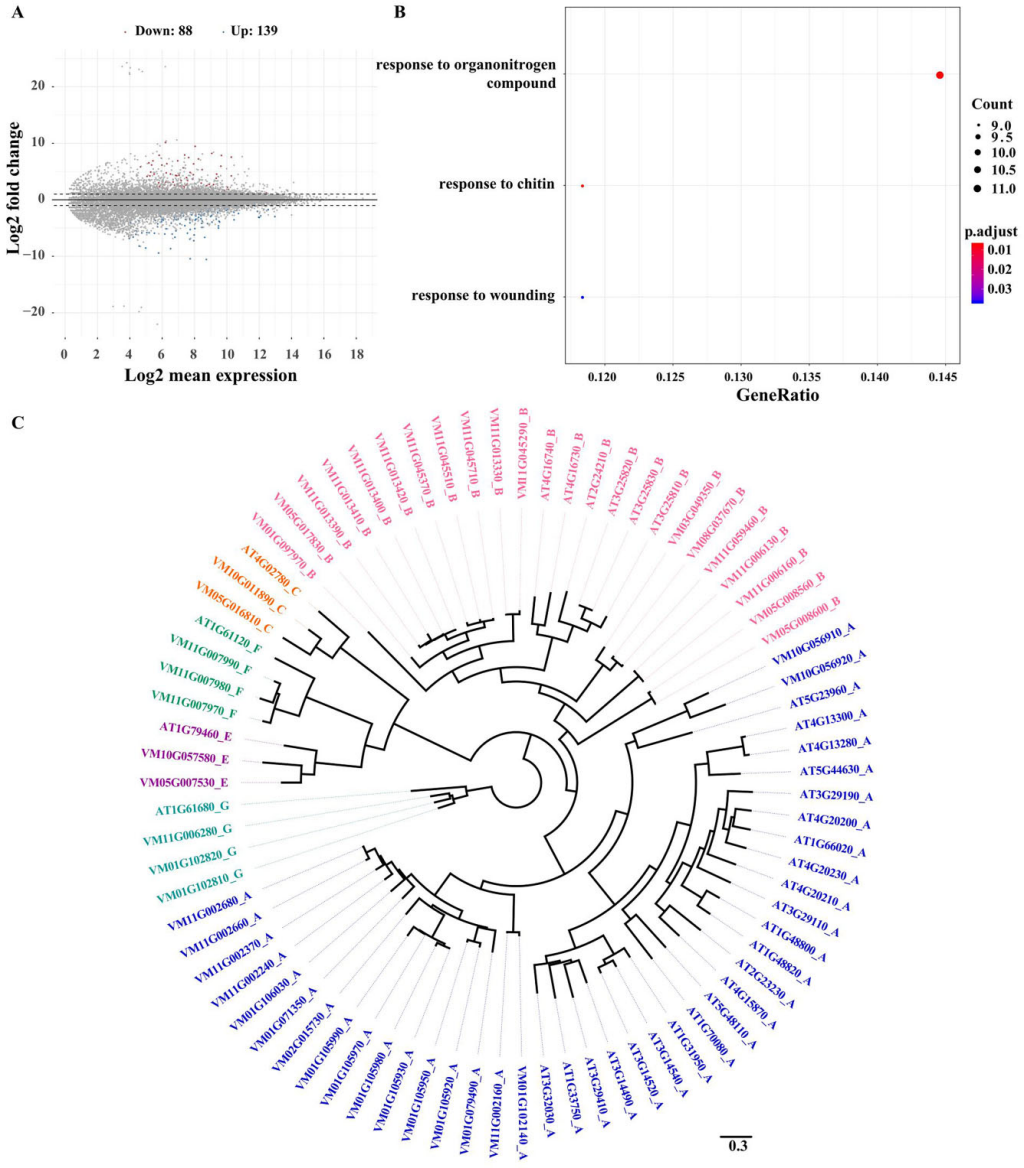
Transcriptome analysis of *V. mangachapoi*. (A) Log-fold difference in differential gene expression between the control group and the drought-stressed group. (B) KEGG enrichment of DEGs between the control and the drought condition in leaves. (C) Phylogenetic analysis of the *TPS* gene family in *V. mangachapoi* compared with similar genes in *Arabidopsis thaliana*.

*Vatica* *mangachapoi* can survive for extended periods without rainfall in seasonally dry zones. The transcriptomes of leaf samples subjected to normal and drought stress conditions were compared with identify genes potentially involved in drought responses. Between 85.30% and 91.70% of the RNA-seq data were mapped to the reference genome (Supplementary Table S10). DESeq2 showed that 227 genes were differentially expressed between the control and the drought-stressed trees, of which 88 were down-regulated and 139 were up-regulated (Fig. 4A). GO enrichment analysis indicated these DEGs were functionally enriched with three GO terms: ‘response to organonitrogen compounds’, ‘response to chitin’, and ‘response to wounding’ (Fig. 4B). Considering that the chitin-triggered immunity pathway is involved in stomatal regulation,^55^ we suspect that this pathway may also regulate drought response in *V. mangachapoi*.

In plants, volatile organic compounds (VOCs) contribute to biotic and abiotic stress resistance.^56^ Some abiotic stresses even lead to the release of VOCs by controlling the biosynthesis, function, and metabolic engineering of plant VOCs (e.g. drought stress alters the amount of volatile compounds emitted from the leaves of apple trees).^56–59^ Previous analyses found that *V. mangachapoi* had several terpene VOCs, including monoterpenes (ocimene, α-pinene, myrcene, and limonene) and sesquiterpenes (α-cedrene).^60^ Here, a total of 46 terpene synthase (*TPS*) genes were identified in the genome of *V. mangachapoi*, including 18 TPS-a, 18 TPS-b, and three TPS-g genes which primarily affect the synthesis of monoterpenes and sesquiterpenes (Fig. 4C and Supplementary Table S11). Interestingly, transcriptomic analysis found that one TPS-a gene (VM11G002680), three TPS-b genes (*VM03G049350*, *VM05G008600*, and *VM11G006130*) and one TPS-g gene (*VM11G006280*) were expressed more by drought-stressed trees than by non-stressed trees (Supplementary Table S12).

## 4. Discussion

*V. mangachapoi* is a species with great economic value both through the production of high-quality timber as well as through the numerous ecosystem services it provides as a keystone species in dipterocarp forests. However, due to the lack of high-quality reference genomes in the Dipterocarpaceae, genomic studies on the molecular basis of wood formation and drought tolerance in *V. mangachapoi* have been limited. Therefore, we used PacBio long reads and Hi-C data to assemble a 456.21 Mb chromosome level genome for *V. mangachapoi* from which 46,811 protein-coding genes were annotated, of which the genome size is consistent with two published genomes of Dipterocarpaceae (*Dipterocarpus turbinatus* Gaertn. f.: 421.2 Mb, *Hopea hainanensis* Merr. et Chun: 434.3 Mb).^54^ The resulting genome is a milestone for the study of dipterocarp forests as it is the first genome of this quality in *Vatica* as well as for the entire Dipterocarpaceae family.

By comparative genomic analyses, a strong collinearity within the genome of *V. mangachapoi* was revealed (Fig. 3A), based on which a WGD event was inferred in this species. WGD could contribute to the increase of the size of plant genomes, moreover, they may expand genetic variation, enhance the complexity of transcriptional regulation, and can prompt lineage divergence and speciation as well.^61^^,^^62^ Given that genome duplication and subsequent diploidization is common among plant lineages,^63^^,^^64^ it is not surprising to find WGD in *V. mangachapoi*. The WGD event is the likely reason for the relatively high level of duplication in BUSCO analysis, and may trigger the expansion and contraction of gene families, thus affecting the environmental adaptation potential of *V. mangachapoi*. In addition, a population outbreak occurred ∼20 MYA, after WGD of the *V. mangachapoi* genome. A shared WGD event between *D. turbinatus* and *H. hainanensis* Chun was reported, and WGD in the common ancestor of Dipterocarpaceae was suggested.^54^ The WGD detected in *V. mangachapoi* is likely the indication of WGD in the common ancestor of the three species. However, the timing of the WGD based on Ks plot analyses of *V. mangachapoi* genome is much younger than the time estimated in Wang et al.^54^ and deserve further investigation. In summary, WGD might contribute not only to the expansion of gene families in *V. mangachapoi* but also to the massive growth of its population size (Fig. 2C).

Previous study has indicated that dipterocarp seedlings were less affected by drought than non-dipterocarp seedlings due to down-regulation of photosynthesis and thus decreasing evapotranspiration in dipterocarp seedlings.^56^ Functional enrichment analysis of DEGs in non-stressed and drought-stressed *V. mangachapoi* trees revealed that genes related to organonitrogen compound metabolism, chitin-triggered immune response, and wounding response were significantly enriched (Fig. 4B). The high expression of chitin-triggered immune genes are generally responsible for mounting a defensive response to fungal infections.^65^ In *A. thaliana*, the cell surface receptor AtCERK1 is homodimerized while bound to chitin, resulting in the activation of innate immunity.^66^ As part of this response, stomatal guard cells would close the stomata, thereby preventing further intrusion of infectious agents into the leaves.^67^ In *Arabidopsis*, a mutation in the *AtRAN1* gene showed not only diminished chitin-induced responses but also increased sensitivity to drought. Therefore, *AtRAN1* might positively regulate drought responses by mediating other stress response genes.^55^ As the possibility of fungal infection in the samples used for the transcriptomic analyses cannot be completely excluded, it is unclear whether the increased expression of chitin-triggered genes was solely the result of drought stress or if fungal infection was involved, as well. However, since drought stress is tightly linked with susceptibility to infection,^55^ genes responsible for stomatal control are likely involved in both abiotic and biotic stress responses. It is possible, then, that drought response pathways in *V. mangachapoi* are largely controlled by chitin-triggered immune response genes that regulate the opening and closing of the stomata, and consequently, reduce photosynthesis and transpiration. Further studies are required to elucidate the underlying mechanisms controlling drought tolerance in *V. mangachapoi* to determine whether unique adaptations have occurred in this pathway.

More than 1,700 VOCs have been identified in plants,^68^ many of which regulate growth and resistance to pathogenic infections in plants.^57^^,^^59^ VOCs released from plants also appear to be involved in abiotic stress resistance.^59^ For example, studies on *Betula pendula* and *Populus tremula* have found that heat stress induced the release of terpenes.^69^ The release of monoterpenoids was also increased under drought stress in *Quercus suber*.^70^^,^^71^ Previous analyses indicated that terpene VOCs in *V. mangachapoi* were mainly comprised of monoterpenes (ocimene, α-pinene, myrcene, and limonene) and sesquiterpenes (α-cedrene).^60^ TPSs are important enzymes responsible for catalysing the MVA and MEP pathways which form the backbones of terpenes.^72^^,^^73^ Of these, TPS-b and TPS-g are mainly involved in producing monoterpenoids, whereas TPS-a genes are involved in the formation sesquiterpenoids. Here, a total of 46 *TPS* genes were identified. Also, we found that the increased expression of *TPS* genes was associated with terpenoid synthesis in *V. mangachapoi* exposed to drought stress (Supplementary Table S12), suggesting that these genes have a putative role in regulating drought tolerance. Further work is needed to understand how terpenes regulate drought responses in *V. mangachapoi*.

## 5. Conclusion

Here, we present the first chromosome-level genome from a species in the Dipterocarpaceae and provide annotations for 46,811 protein-coding genes with follow-up expression data for many of these genes. Repeats were also identified, of which LTRs was the most abundant transposable element, accounting for 39.44% of the genome. A genome-wide duplication event likely occurred in *V. mangachapoi*, impacting both gene count and population size. Comparative transcriptome analysis showed that DEGs in *V. mangachapoi* were mainly involved in responses to organonitrogen compounds, chitin-triggered immunity, and responses to wounding which may affect drought resistance in this species. The genomic data provided in this article will not only enable further molecular studies in *Vatica* and Dipterocarpaceae but also provide resources for breeding drought-resistant dipterocarp species that can be replanted in drought-afflicted areas.

## Supplementary data

Supplementary data are available at DNARES online.

## Supplementary Material

dsac005_Supplementary_Data
