# Supplementary figures and images for: A chromosome-scale genome and transcriptomic analysis of the endangered tropical tree Vatica mangachapoi (Dipterocarpaceae)
Source: DNA Res. 2022 Feb 16;29(2):dsac005. doi: 10.1093/dnares/dsac005 (PMC8882376; doi:10.1093/dnares/dsac005)

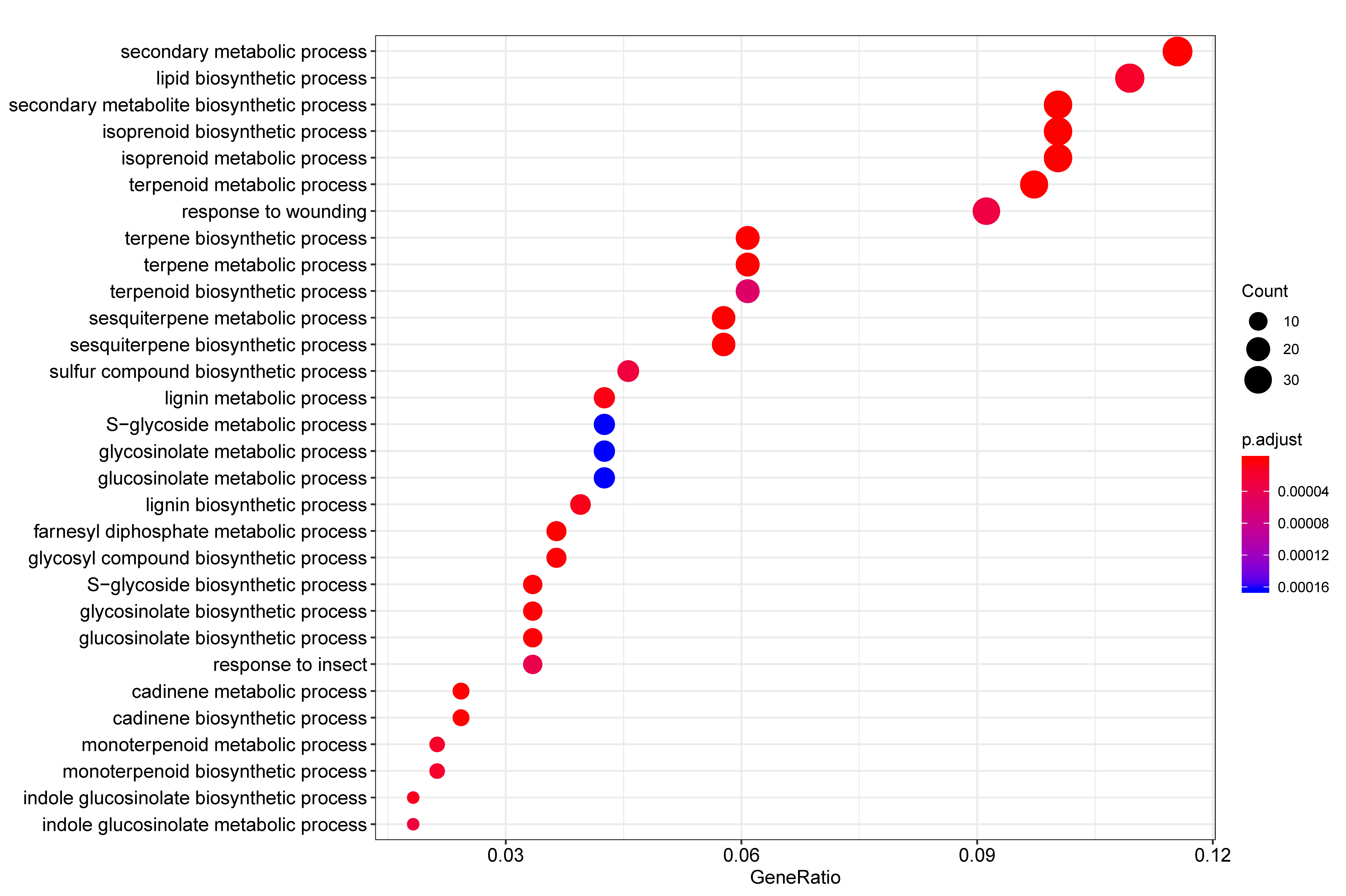

Supplement: dsac005_Supplementary_Data [file dsac005_supplementary_data.zip › Figure S2. GO enrichment of contacted gene family.jpg]

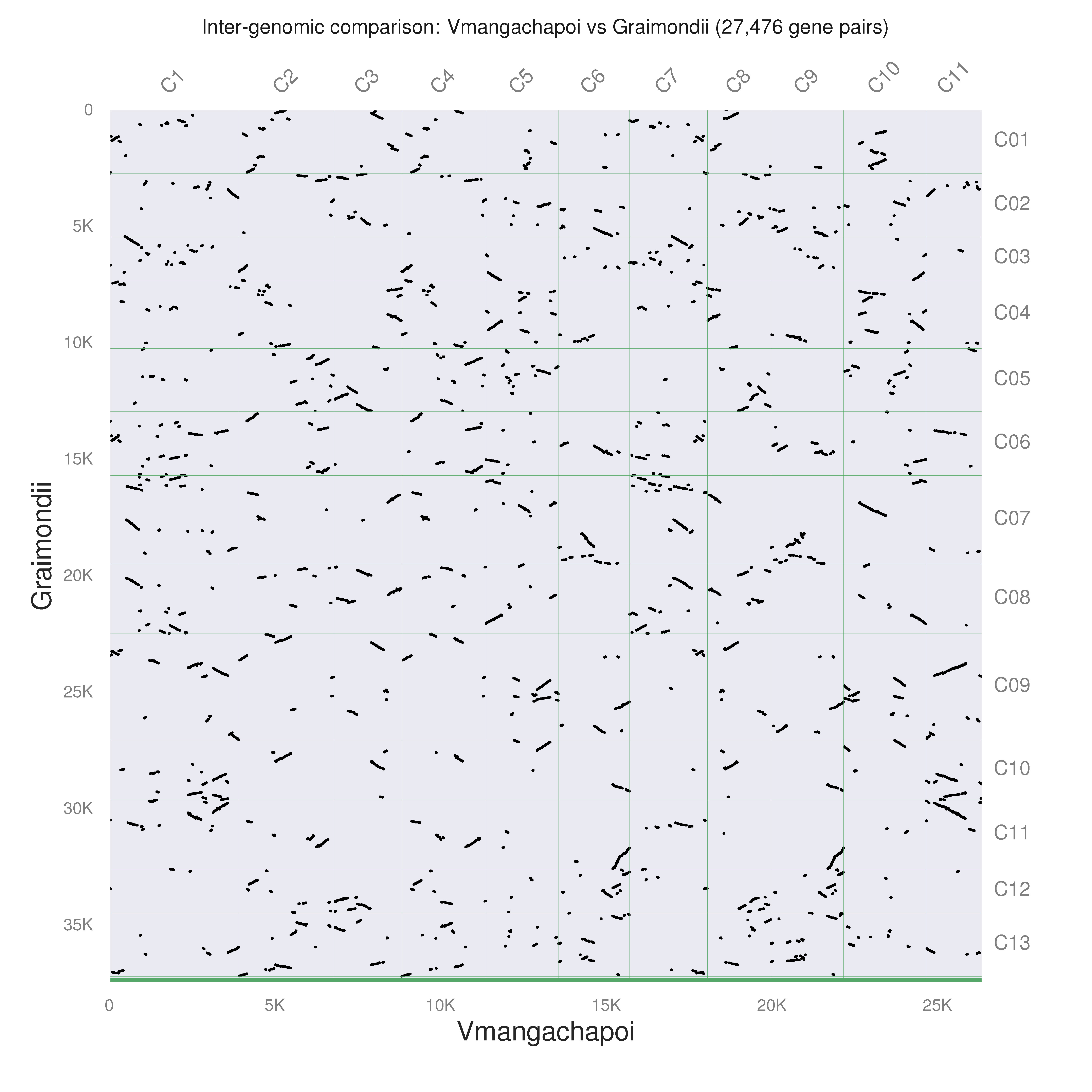

Supplement: dsac005_Supplementary_Data [file dsac005_supplementary_data.zip › Figure S3. Collinearity between V. mangachapoi and G. raimondii.jpg]

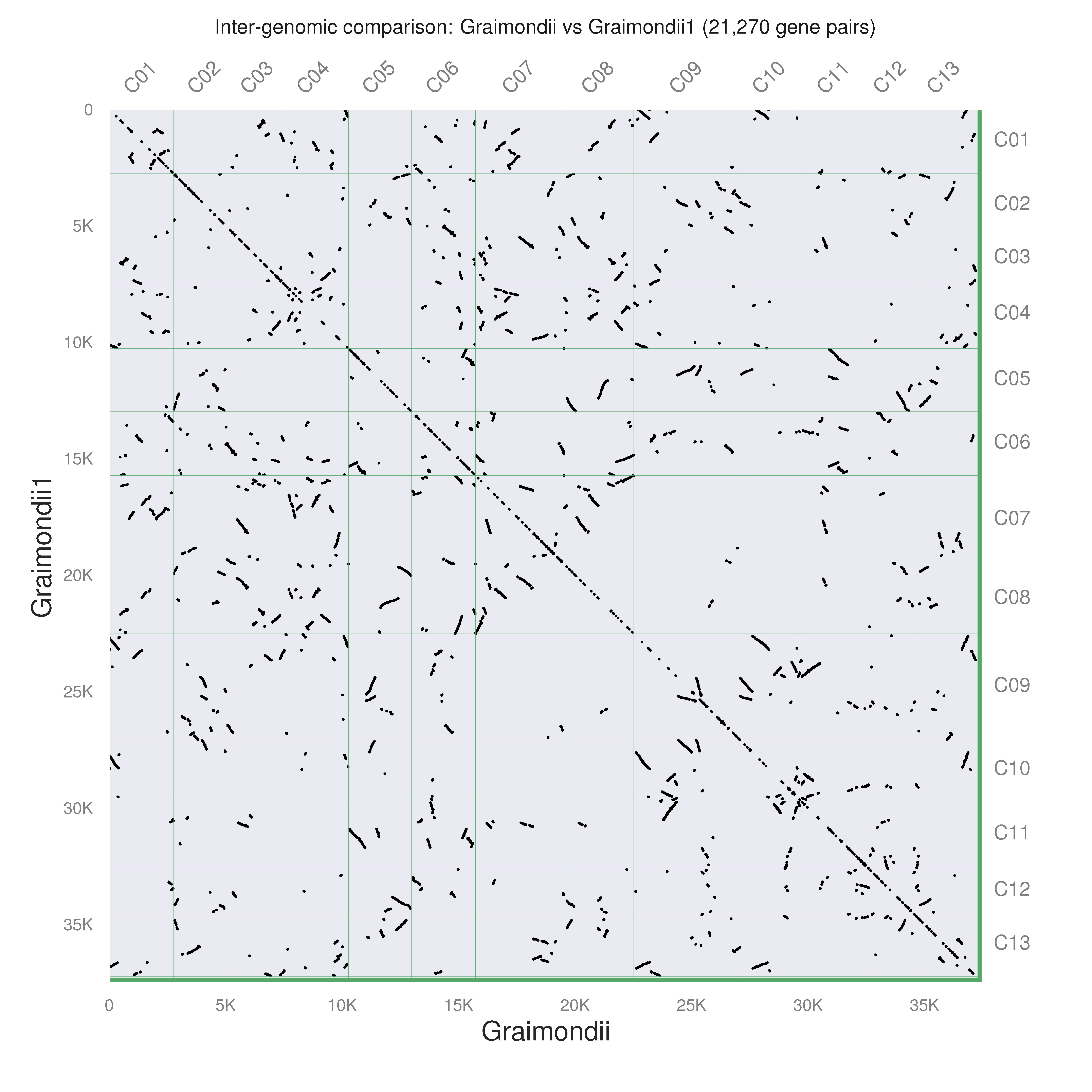

Supplement: dsac005_Supplementary_Data [file dsac005_supplementary_data.zip › Figure S4. Collinearity within G. raimondii.jpg]

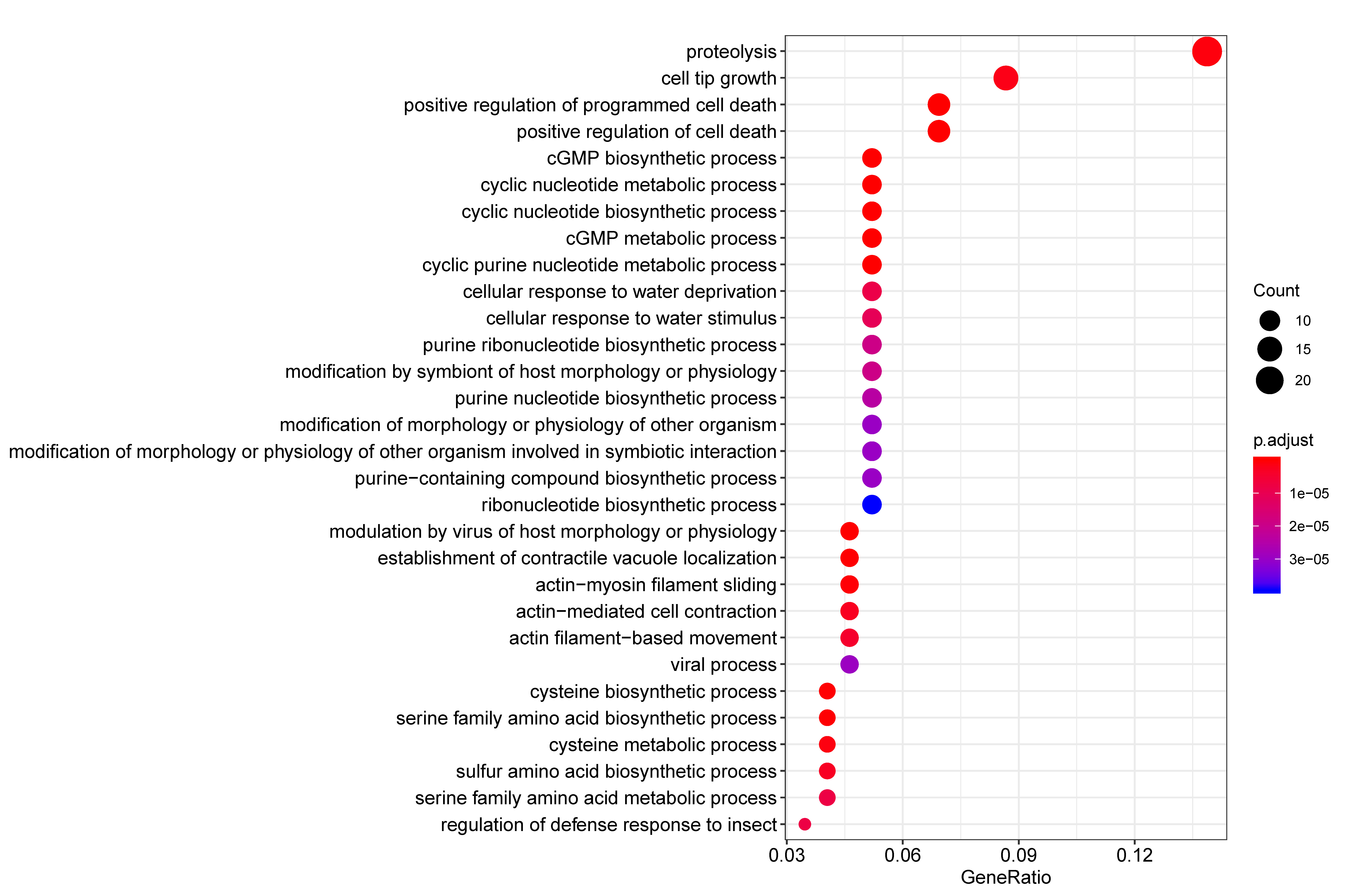

Supplement: dsac005_Supplementary_Data [file dsac005_supplementary_data.zip › Figure S1. GO enrichment of expanded gene family.jpg]
